# Do Aerial Nitrogen Depositions Affect Fungal and Bacterial Communities of Oak Leaves?

**DOI:** 10.3389/fmicb.2021.633535

**Published:** 2021-04-16

**Authors:** Luigimaria Borruso, Alessia Bani, Silvia Pioli, Maurizio Ventura, Pietro Panzacchi, Livio Antonielli, Francesco Giammarchi, Andrea Polo, Giustino Tonon, Lorenzo Brusetti

**Affiliations:** ^1^Department of Science and Technology, Free University of Bozen-Bolzano, Bolzano, Italy; ^2^School of Life Sciences, University of Essex Colchester Campus, Essex, United Kingdom; ^3^Department of Bioscience and Territory, University of Molise, Pesche, Italy; ^4^Center for Health & Bioresources, AIT Austrian Institute of Technology, Vienna, Austria

**Keywords:** microbial communities, *Quercus petraea*, temperate forest, Alps, forest ecology

## Abstract

The amount of nitrogen (N) deposition onto forests has globally increased and is expected to double by 2050, mostly because of fertilizer production and fossil fuel burning. Several studies have already investigated the effects of N depositions in forest soils, highlighting negative consequences on plant biodiversity and the associated biota. Nevertheless, the impact of N aerial inputs deposited directly on the tree canopy is still unexplored. This study aimed to investigate the influence of increased N deposition on the leaf-associated fungal and bacterial communities in a temperate forest dominated by Sessile oak [*Quercus petraea* (Matt.) Liebl.]. The study area was located in the Monticolo forest (South Tyrol, Italy), where an ecosystem experiment simulating an increased N deposition has been established. The results highlighted that N deposition affected the fungal beta-diversity and bacterial alpha-diversity without affecting leaf total N and C contents. We found several indicator genera of both fertilized and natural conditions within bacteria and fungi, suggesting a highly specific response to altered N inputs. Moreover, we found an increase of symbiotrophic fungi in N-treated, samples which are commonly represented by lichen-forming mycobionts. Overall, our results indicated that N-deposition, by increasing the level of bioavailable nutrients in leaves, could directly influence the bacterial and fungal community diversity.

## Introduction

In the last decades, human activity has dramatically altered the nitrogen (N) cycle on Earth, doubling the annual global production of reactive nitrogen (Galloway et al., 2004). In Europe and the United States, N deposition increased from 5 to 50 times more than in the pre-industrial era (Kamble et al., 2013). This trend is foreseen to continue in temperate regions, primarily due to animal-production systems and fuel combustion (Erisman et al., 2014). Nitrogen emissions impact the air quality, increasing tropospheric ozone, smog and particulate matter (Erisman et al., 2014). Further, freshwater systems can be affected negatively in terms of water quality, promoting the increase of eutrophication levels and acidity (Vitousek et al., 1997). With regards to the terrestrial ecosystems, acidification is a common consequence of N deposition along with soil deterioration due to the loss of nutrients, including calcium and potassium (Bergström and Jansson, 2006; Erisman et al., 2014). Besides, it has been hypothesized that the alteration of natural N concentrations can affect biogeochemical cycles, mainly of carbon (C) (Fornara and Tilman, 2012). Previous studies carried out in urban, agricultural (Ramirez et al., 2010; Zeng et al., 2016; Zhou et al., 2016) and natural environments, including forests (Entwistle et al., 2013) and alpine tundra (Bowman et al., 2008), have shown that N changes can affect the microbial diversity and inhibit the microbial biomass growth (Ramirez et al., 2010; Zeng et al., 2016).

Different studies, including field-scale N-manipulating experiments, have explored N deposition effects on the microbial communities in soil or other environmental matrices (Ramirez et al., 2010; Zhou et al., 2016). However, only a few of these studies have used above-canopy N fertilization to simulate an increased N deposition (Zhang et al., 2015; Shi et al., 2016). Specifically, these studies considered only specific groups of prokaryotes (soil ammonia-oxidizing archaea and bacteria) or evaluated the bacterial biomass changes (Zhang et al., 2015; Shi et al., 2016). The authors found a decline of ammonia-oxidizers and the microbial biomass in the phyllosphere of treated samples after N application. Nevertheless, both studies did not consider the effects on the leaf-associated fungal and bacterial communities.

With this regard, we intended to analyze the short-term effects of the direct canopy N-deposition on the leaf-associated microbial communities. In detail, we wanted to answer to two experimental questions: (i) Do the N-depositions modify the leaf chemical features? (ii) Are leaf-associated microbial communities, namely bacteria and fungi, resistant when subjected to the N-deposition?

## Materials and Methods

### Experimental Design and Sampling

The experiment has been conducted in a sessile oak [*Quercus petraea* (Matt.) Liebl.] stand located in the Monticolo forest (Autonomous Province of Bolzano, Italy) (46°25′35′N; 11°17′55′E). A more detailed description of the forest site can be found in Giammarchi et al. (2020). Six circular plots (12 m radius) were settled in the forest in 2014, where no other manipulative experiment had been previously performed. The plots were established on a relatively small area (about 200 m of length), so that the conditions were homogeneous in terms of vegetation, aspect, and soil. Three plots received aerial fertilization above the canopies (N-treated), while other three plots received only sterile water (Control). Plots were arranged in a completely randomized design in the experimental site. Plots were separated by at least 9 m of buffer distance and their topographical location was chosen to avoid contamination from the treated to the control ones. Treatments were performed in days without wind to avoid any drifting effect. The fertilization treatments delivered a total of 20 kg N ha^–1^ y^–1^, which is more than three times higher than background atmospheric bulk N deposition (Marchetti et al., 2003). Nitrogen was provided as a NH_4_NO_3_ solution (4.3 g l^–1^), with 5 monthly applications from May 2015 until September 2015. The water provided with the treatments amounted to 210 l H_2_O plot^–1^ yr^–1^ in total, equivalent to a precipitation of 0.46 mm, which is negligible compared to the average annual precipitation for the region (around 800 mm) (Marchetti et al., 2003).

Aerial treatments were applied above the canopy, at 15–18 m height, depending on trees height in each single plot, using rotating sprinklers (Rain Bird SNC, Aix-en-Provence, France) mounted on telescopic masts (Fireco S.R.L., Gussago, Brescia, Italy) installed in the center of the plots, and one portable motor pump (Officine Carpi S.R.L., Poviglio, Reggio Emilia, Italy). The sprinkle provided a uniform spray diameter of ∼12 meters on the canopy tops, covering the whole plot area. A different irrigation system (tubing and sprinkler) was used for the treatment of each plot. Irrigation systems were left in the field between the treatment events to avoid contamination with exogenous bacteria.

In each of the six plots, 15 days after the last fertilization, leaves from three trees were sampled. For each of the 18 trees, 20 leaves were randomly collected (five leaves from each cardinal point within each sampled tree), at a height of about 8 m. Leaves were placed in sterile bags. Leaves were processed within 4 h of sampling and subsampled as follows: 10 leaves from each tree were ground to a fine powder under liquid nitrogen using a sterile mortar and pestle and stored at –20°C; the others (n. 10) were used for the Leaf Mass Area analysis.

### Leaves Characterization

Samples for the Leaf Mass Area analysis were oven-dried for 72 h at 60°C. Total Carbon C and Nitrogen N were determined using an elemental analyzer (Flash 2000, Thermo Fisher Scientific). Weight and leaf area were measured considering 10 leaves for each sample using a LI-3000 leaf area meter (LI-COR, Lincoln, Nebraska, United States). Leaf mass per area (LMA) was calculated as the weight of leaves divided by the total leaf area.

### DNA Extraction

DNA was extracted from ∼0.25 g of powder using the PowerPlant^®^ Pro DNA Isolation Kit according to the user’s manual (Qiagen, Arcore, Italy). Extracted DNA was stored at –20°C. DNA concentration was evaluated via fluorimetry using the PicoGreen quantification.

### Next Generation Sequencing and Bioinformatics Analysis of Bacterial 16S rRNA Genes and Fungal ITS Regions

A nested PCR approach was used to avoid co-amplification of chloroplast and mitochondrial ribosomal genes, as reported in Mitter et al. (2017). The first amplification was done with primers 799F (5′-AACMGGATTAGATACCCKG-3′) and 1392R (5′-AGGGCGGTGTGTRC-3′) (Chelius and Triplett, 2001). This primer pair allows exclusion of the chloroplast 16S rRNA. The amplification results in co-amplification of bacterial and mitochondrial ribosomal genes. The band containing the PCR-product of bacterial 16S rRNA was excised. The second amplification was performed on the purified DNA using primers 799F and 1175R modified with the required Illumina sequencing adaptors. Fungal ITS region was amplified using primers ITS31_NeXTf 5′-CATCGATGAAGAACGCAG-3′ and ITS4_NeXTr 5′-TCCTCCGCTTATTGATATGC-3′ (Tedersoo et al., 2015), with the required Illumina sequencing adaptors, according to the protocol of Borruso et al. (2018). The PCR products were analyzed using the Illumina MiSeq platforms (2 × 300 bp) paired-end sequencing technology, following the standard protocols of the company STAB Vida Lda. (Caparica, Portugal). Raw data derived from the 16S rRNA gene and fungal ITS region Illumina runs were processed independently according to the following pipeline. Raw data quality was checked in FastQC and reads were screened for PhiX contamination using Bowtie 2.2.6 (Langmead and Salzberg, 2012). A Bayesian clustering for error correction was applied (Nikolenko and Alekseyev, 2011) before merging the PE reads using PEAR 0.9.6 (*p* < 0.001) (Zhang et al., 2014). Forward and reverse primers were then stripped from merged reads employing Cutadapt 1.8.3 (Martin, 2013) and quality filtering performed in USEARCH v8.0 (maximum expected error = 0.5) (Rognes et al., 2016). METAXA2 v2.2 was used to check SSU ribosomal reads and to verify the 16S rRNA V5-V7 region of bacterial sequences. ITS reads were processed with ITSx v1.1 to verify and to target the presence of fungal ITS2 sequences (Bengtsson-Palme et al., 2013). Targeted reads were labeled according to the sample name of origin and combined in QIIME 1.9.1 (Caporaso et al., 2010). Sequences were dereplicated, sorted and clustered at 97% of similarity using VSEARCH 1.1.1 (Rognes et al., 2016). Chimeras were removed, adopting a *de-novo*-based approach, as routine of the above-mentioned tool. An optimal global alignment was applied afterward in VSEARCH and a BIOM table generated. Taxonomy assignment was performed employing the naïve Bayesian RDP classifier v2.10 in QIIME using SILVA release 132 (Quast et al., 2013) and UNITE 8.0 (Abarenkov et al., 2010) as reference databases for bacterial and fungal sequences, respectively.

Alpha and beta diversity analyses were conducted on data rarefied to 29,688 and 99,904 sequences for fungi and bacteria, respectively. Fungal data were parsed against the FunGuild (v1.0) database to assign putative functional guilds to Operational Taxonomic Units (OTU) groups (Nguyen et al., 2015). All sequences have been submitted to the EMBL/NCBI/DDBJ under accession numbers from ERS3396975 to ERS3397010.

### Statistical Analysis

Statistical analyses were performed using multi packages of R software (R Core Team, 2017). Alpha diversity was investigated via Observed OTUs and Simpson indices. The data normality was tested via Shapiro-Wilk. The Observed OTUs and Simpson indices were tested for statistical difference between N-treated and control samples through the Welch Two Sample *t*-test for bacteria, while for fungi Wilcoxon Rank Sum test has been applied since the data were not normally distributed. Beta dispersion was tested for fungal and bacterial using the “vegan::betadisper” function. Beta dispersion was calculated to test if the groups had the same centroids and heterogeneity. PERMANOVA was applied both on OTU level and on the genus level to test the treatment’s effect. Ordination plots were created applying Constrained Analysis of Principal Coordinates (CAP) based on Bray Curtis distance at the genus level for both bacteria and fungi (“capscale” function). Finally, to test the significance of the correlation between the fungal and bacterial CAP analysis, a Procrustes was applied.

Indicator taxa analysis was conducted by fitting taxa, summarized at genus level, in a Poisson-based generalized linear model followed by Bayesian analysis with Markov chain Monte Carlo (MCMC.OTU package at https://cran.r-project.org/web/packages/MCMC.OTU/index.html for more details). Genera showing *p* < 0.05 after FDR were selected and displayed in the boxplots.

Further, Generalized Linear Models (GLMs) were created and then tested with ANOVA analysis with the mvabund package library in R to analyze if the putative functionality guilds of fungi were statically influenced by the treatment (Wang et al., 2012).

## Results

### Leaf Functional Traits and Microbial Community Composition of the N-Treated and Untreated Leaves

The C/N ratio, N concentration, C concentration, and LMA were not significantly different between N-treated and control samples (Table 1).

**TABLE 1:** Average ± standard deviation, *t*-value, and *p*-value for the leaf functional traits of control and N-treated samples.

	**Control**	**N-treated**	***t*-value**	***p*-value**
N (%)	1.85 ± 0.07	1.9 ± 0.3	0.53	0.29
C (%)	46.0 ± 1.0	46.7 ± 0.4	1.34	0.10
C/N	25 ± 1.00	25 ± 4	0.05	0.48
(#) LMA (g m^–2^)	84 ± 18	83 ± 9	-0.10	0.46

**(#) Leaf Mass Area.**

After bioinformatics pipelines and quality filtering, a total of 3,045,338 bacterial reads and 1,960,578 fungal reads were found (Supplementary Tables 1, 2). The number of OTUs assigned as bacteria and fungi had an average of 3,290 and 488, respectively (Supplementary Tables 1, 2). No taxonomically unassigned OTUs were detected in the filtered bacterial data; differently unassigned fungal OTUs represented 22% of the total number of fungal OTUs but only accounting for 7% of the whole fungal reads.

Sequences were taxonomically assigned to 19 bacterial phyla, 38 classes, 68 orders, 146 families, 270 genera, while for fungi: 3 phyla, 19 classes, 65 orders, 148 families, and 352 genera. Concerning Bacteria, the most abundant Phyla in both treated and control samples were Proteobacteria, Bacteroidetes, Actinobacteria, Acidobacteria, and Firmicutes (Supplementary Figure 1A).

At the genus level, control samples were characterized by the following bacterial indicator taxa: *Bifidobacterium*, *Gilliamella*, *Lactobacillus*, and *Streptococcus* (Supplementary Figure 2A and Supplementary Table 3). Conversely, *Leuconostoc*, *Zymobacter*, and Candidatus *Carsonella* were the genera that better reflected the nitrogen-treated samples (Supplementary Figure 2B and Supplementary Table 3).

The fungal community across all samples was dominated by members affiliated with the phylum Ascomycota, followed by Basidiomycota (Supplementary Figure 1B). Fungal indicator genera characterizing control samples were: *Curreya*, *Endomelanconiopsis*, *Hypotrachyna*, *Naevala*, *Rhinocladiella*, *Roussoella*, and *Saccharomyces* (Supplementary Figure 2B and Supplementary Table 4). On the contrary, *Microcera* and *Polyscytalum* were the fungal indicators for nitrogen-treated samples (Supplementary Figure 2B and Supplementary Table 4).

### Alpha and Beta Diversity

**FIGURE 1:**
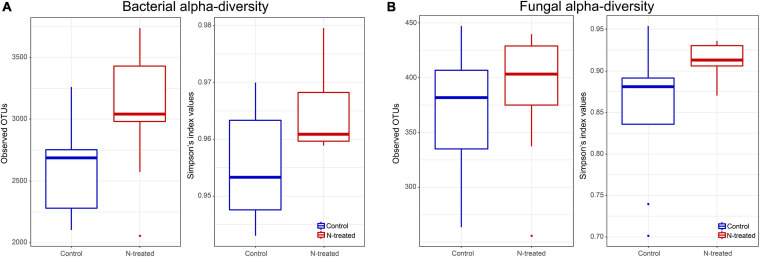
Box plots representing the average and standard deviation of observed operational taxonomic units (OTUs) and Simpson indices of bacterial **(A)** and fungal **(B)** communities.

**FIGURE 2:**
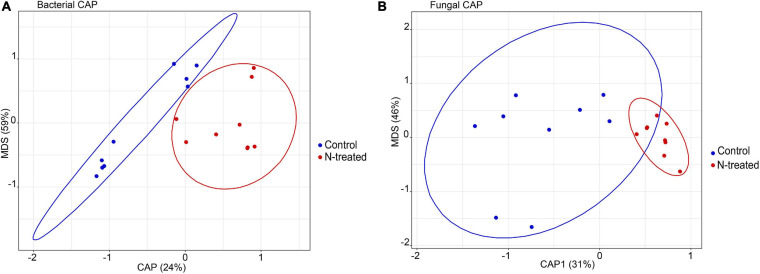
Constrained Analysis of Principal Coordinates (CAP) ordination plot on bacterial **(A)** and fungal genera **(B)**. In blue the control samples, in red the N-treated samples.

For the bacterial community, Observed OTUs (*t* = –2.17, *d.f.* = 14.73, *p* = 0.04) and OTU Simpson’s diversity (*t* = –2.38, *d.f.* = 14.66, *p* = 0.03) were higher in N-treated samples than control samples (Figure 1A). Contrarily, fungal Observed OTUs and Simpson’s diversity were not different between N-treated and control samples (Figure 1B). Beta dispersion of bacterial and fungal communities revealed that N-treated samples and control samples had the same centroid (bacterial PERMDISP: *p* = 0.82, *F* = 0.07; fungal PERMDISP: *p* = 0.64, *F* = 0.24). Pairwise comparison showed that the groups do not have statistically different dispersion (bacterial *p*-value: below diagonal = 0.82, above diagonal = 0.81; fungal *p*-value: below diagonal = 0.63, above diagonal = 0.63), confirming that there was no difference in community heterogeneity across treatments. PERMANOVA test applied to bacterial OTU 97% and on genus level revealed no statistical differences between treated and control samples (PERMANOVA: OTU 97%, *R*^2^ = 0.7, *p* = 0.21; OTUs at genus level *R*^2^ = 0.1, *p* = 0.07). At genus level, the canonical analysis of principal coordinates (CAP; Anderson and Willis, 2003) of bacterial data discriminated between treated and control samples (*F* = 1.84, *p* = 0.04) (Figure 2A). Fungal samples were clearly differentiated according to treatment (PERMANOVA: OTU 97%, *R*^2^ = 0.11, *p* = 0.01; OTUs at genus level *R*^2^ = 0.13, *p* = 0.003). CAP on fungal genera validated these results grouping the treated and control samples into two clusters (Figure 2B), with significant differences among experiments (*F* = 1.63, *p* = 0.008) (Figure 2B). A large degree of congruence between the bacterial and fungal genus distribution pattern based on a matrix comparison using Procrustes analysis was found (*m*^2^: 0.64, *R*^2^: 0.60, *p*: 0.001).

### Fungal Predictive Functional Analysis

Predictive functional and ecological features of fungal populations were investigated using the FunGuild analysis applied to the overall fungal OTUs. The most abundant ecological guild was “Plant-Pathogen,” while the “Fungal-Parasite,” “Lichenized,” and “Endophyte” were less represented. Concerning the fungal growth morphology, the facultative yeast-like and microfungal were the most frequent, followed by “Thallus” and “Yeasts.” Further, the “Pathotrophy” trophic mode was more abundant than “Saprotrophs” and “Symbiotrophs.” Regarding the differences between treatments, fungi characterized by “Symbiotrophic” behavior, associated with lichens and characterized by a thallus-like growth, increased in N-treated samples (Table 2).

**TABLE 2:** Fungal Guild, Growth Morphology and Trophic Mode information described in accordance with the FunGuild software.

**Fungal guild**	**Control**	**N-treated**	***p*-value**
Endophyte	157 ± 58	126 ± 37	0.70
Fungal-parasite	536 ± 290	699 ± 401	0.66
Lichenized	263 ± 67	515 ± 225	0.02*
Plant-pathogen	4,490 ± 2,600	4,950 ± 1,850	0.69

**Growth morphology**	**Control**	**N-treated**	***p*-value**

Facultative-yeast	1,110 ± 410	1,150 ± 519	0.85
Microfungus	2,610 ± 2,180	2,650 ± 1,240	0.93
Thallus	263 ± 67	367 ± 225	0.02*
Yeasts	203 ± 188	207 ± 86	0.98

**Trophic mode**	**Control**	**N-treated**	***p*-value**

Pathotroph	4,500 ± 2,600	4,960 ± 1,840	0.96
Saprotroph	1,590 ± 966	1,100 ± 375	0.39
Symbiotroph	423 ± 93	645 ± 229	0.04*

Values represent the mean of reads (considering the rarefied OTU table) of all the samples in each treatment with standard deviation. The p-values are calculated with mvabund package. The asterisk (*) indicates the significant difference between Control and N-treated samples.

## Discussion

The first research question asked whether N-depositions were able to modify leaf functional traits, while the second question investigated if leaf-associated microbial communities had been affected in their alpha or beta-diversity. Leaf functional traits (i.e., LMA, C/N ratio, N and C concentration) did not show differences between controls and N-treated samples. This could be due to the short experimental duration and the slow physiological tree response and adaptation. Giammarchi et al. (2020), on the same site, did not find significant differences, caused by the treatment in some leaf functional traits in the successive 3 years. To this extent it has been shown that prolonged N-deposition could be associated to an increase of N foliar concentration over time (Hobbie, 2015). On the other hand, we found a significant influence of the N-deposition on the microbial community diversity and ecological features, confirming that bacteria are more sensitive to an environmental disturbance (Borruso et al., 2015; Cong et al., 2015). Specifically, the N-deposition increased bacterial alpha-diversity and slightly affected bacterial beta-diversity. These findings are coherent with what found in forest soils subjected to prolonged N-depositions (Zhou et al., 2017).

The increase in bacterial alpha-diversity is somewhat unexpected as other studies found a general decrease in leaf and soil bacterial diversity after N fertilization (Manching et al., 2014; Li et al., 2016). However, differently from soil, leaves are an oligotrophic environment for microorganisms, with limitations both for carbon and nitrogen availability (Kim et al., 2011). Thus, the supplying of N could have promoted an increased bacterial diversity (Brankatschk et al., 2011) as evidenced by an increment of the abundance of some indicator species, such as the *Leuconostoc*, which is associated to copiotrophic environments (McEniry et al., 2008).

Nevertheless, the increase in N availability did not have strong effects on beta-diversity bacterial communities, indicating that a more extended treatment period or higher amount of applied N may be necessary for a clear biological response (as also reported for the leaf traits).

Alternatively, it is possible that highly diverse microbial communities are more likely to contain taxa with complementary response traits, increasing the chance to answer to changing environmental conditions (Bringel and Couée, 2015). With these regards, bacterial communities could rapidly recover after a disturbance, such as N addition (Shade et al., 2012).

According to the indicator species analysis, several taxa were related to undisturbed environmental conditions. We reported *Gilliamella* genus to be an indicator of untreated plots. This genus includes honeybee gut symbiont species, such as *G. apis* that can provide beneficial functions for the host insect like nutrient synthesis, digestion and provision of disease resistance (Kwong and Moran, 2013). The preference of *Gilliamella* for undisturbed conditions may have relevant implications for maintaining key ecosystem processes. Therefore, further studies on pollinator insects are required to unveil the relationships between host insects and their gut biota in response to N fertilization.

On the other hand, other bacterial genera were found to be significantly related to fertilized conditions. For instance, “Ca. *Carsonella*” is a widespread obligate phytopathogenic symbiont of psyllids (Katsir et al., 2018), initially discovered in association with *Celtis occidentalis* L. The reason why oak tree leaf tissues host *Carsonella* is an open question. Possibly, it could have a different behavior than phytopathogens. In fact, Raddadi et al. (2010) discovered a new bacterial species named “Ca. *Liberibacter europaeus*,” in pear tissues, belonging to a recognized group of phytopathogenic bacteria. They also found that it apparently did not cause any disease to plants, probably dwelling as an endophyte (Raddadi et al., 2010). According to their findings, we hypothesize that aerial N-fertilization could induce primary insect symbionts surveillance over the plant leaf surface, with unexplored ecological and, most likely, phytopathological consequences.

Unlike what we have observed for bacteria, N deposition did not affect fungal alpha diversity but influenced its beta-diversity. Fungal communities in fertilized samples clustered differently from control samples. In detail, we found an increase of symbiotrophic fungi in N-treated samples. This group of fungi is not correlated with plant pathogeny and receives nutrients by exchanging resources with the host cells (Petrini, 1991; Nguyen et al., 2015). It has been reported that symbiotic relationships let microorganisms to better tolerate environmental changes (Denton and Karlén, 1973). Taxa with a lichen-forming mycobiont strategy characterized the majority of symbiotrophic. Fungi associated with lichen symbiosis are usually combined with green algal species and a smaller number of Cyanobacteria (Grube and Wedin, 2016). A possible explanation of the increase of lichen-associated fungi could be related to a direct increase of algae cells on the leaf surface due to the increased N availability. Since C and N are the main limiting factors for the growth of microorganisms in leaves and considering that fungi and most bacteria are not photosynthetic organisms, algae could be more facilitated in leaf colonization due to their autotrophic metabolism (Lindow and Brandl, 2003; Vorholt, 2012). Consequently, N deposition could have directly promoted an increase of algal cell number and, indirectly, an increase of fungal cell number with a lichen-forming strategy.

## Conclusion

In conclusion, we found that N-deposition could directly influence the bacterial community alpha-diversity, increasing the level of nutrients bioavailable for microorganisms in leaves, considered so far as an oligotrophic environment. Moreover, we found that N-deposition could indirectly affect fungal beta-diversity, maybe due to the increase of available organisms capable of the symbiontic behavior. Further investigations are needed to shed new light on the potential functional modification of the leaf-associated microbial communities, especially related to carbon sequestration due to autotrophic cell growth.

## Data Availability Statement

The datasets presented in this study can be found in online repositories. The names of the repository/repositories and accession number(s) can be found in the article/Supplementary Material.

## Author Contributions

LBo: performing experiments, conducting the work, writing of the manuscript, design of the work, analysis, interpretation of data for the work, responsible for the integrity of the work as a whole, and final approval of the version to be published. AB: performing experiments, conducting the work, analysis, and critically revising the final approval of the version to be published. SP: performing experiments, conducting the analysis, and critically revising the final approval of the version to be published. MV, PP, FG, and AP: performing experiments, conducting the work, and analysis. LA: conducting the analysis. GT: interpretation of data for the work and critically revising the final approval of the version to be published. LBr: design of the work, interpretation of data for the work, responsible for the integrity of the work as a whole, final approval of the version to be published, and ensuring that questions related to the accuracy or integrity of any part of the work are appropriately investigated and resolved. All authors contributed to the article and approved the submitted version.

## Conflict of Interest

The authors declare that the research was conducted in the absence of any commercial or financial relationships that could be construed as a potential conflict of interest.
