# Supplementary material for: Do Aerial Nitrogen Depositions Affect Fungal and Bacterial Communities of Oak Leaves?
Source: Front Microbiol. 2021 Apr 16;12:633535. doi: 10.3389/fmicb.2021.633535 (PMC8085328; doi:10.3389/fmicb.2021.633535)
Supplement: Supplementary file 1 [file Data_Sheet_1.docx]

**SUPPLEMENTARY INFORMATION**

**Do aerial nitrogen depositions affect leaf-associated fungal and bacterial communities of oak leaves?**

**Luigimaria Borruso^1^, Alessia Bani^1,2^, Silvia Pioli^1^, Maurizio Ventura^1^, Pietro Panzacchi^1,3^, Livio Antonielli^4^, Francesco Giammarchi^1^, Andrea Polo^1^, Giustino Tonon^1^ and Lorenzo Brusetti^1*^**

*^1^ Department of Science and Technology, Free University of Bozen-Bolzano, Bozen-Bolzano, Italy, ^2^ School of Life Sciences, University of Essex, Wivenhoe Park, Colchester, Essex CO4 3SQ, UK, ^3^ Department of Bioscience and Territory, University of Molise, Pesche (IS), Italy, ^4^ Center for Health & Bioresources, AIT Austrian Institute of Technology, Vienna, Austria*

^*^Corresponding author: lorenzo.brusetti@unibz.it

**Contents**

1. Supplementary Tables 2
2. Supplementary Figures 6

**1. SUPPLEMENTARY TABLES**

**Table S1**: Bacterial and fungal sequences and operational taxonomic units (OTUs)obtained per each sample after filtering.

|  | | | | |  |
| --- | --- | --- | --- | --- | --- |
|  | Quality filtered bacterial reads | | Quality filtered fungal reads | | |
| Samples name | # reads | # OTUs | # reads | # OTUs | |
| 2.C.056 | 225,461 | 3,018 | 214,701 | 592 | |
| 2.C.058 | 178,999 | 3,260 | 132,826 | 508 | |
| 2.C.094 | 161,604 | 3,460 | 114,718 | 530 | |
| 3.N.129 | 197,820 | 4,484 | 233,043 | 733 | |
| 3.N.132 | 193,269 | 4,147 | 162,641 | 628 | |
| 3.N.141 | 178,969 | 3,869 | 152,620 | 587 | |
| 4.C.160 | 128,437 | 2,912 | 102,051 | 490 | |
| 4.C.163 | 116,227 | 2,186 | 112,199 | 616 | |
| 4.C.185 | 170,304 | 3,723 | 38,550 | 348 | |
| 6.N.258 | 180,914 | 3,494 | 114,574 | 467 | |
| 6.N.262 | 195,526 | 3,662 | 121,924 | 520 | |
| 6.N.274 | 199,012 | 3,658 | 61,652 | 472 | |
| 7.C.313 | 106,919 | 2,313 | 100,980 | 313 | |
| 7.C.326 | 99,904 | 2,686 | 99,359 | 583 | |
| 7.C.339 | 125,669 | 2,424 | 29,688 | 289 | |
| 8.N.381 | 162,489 | 2,377 | 44,224 | 272 | |
| 8.N.407 | 201,534 | 3,141 | 78,606 | 381 | |
| 8.N.409 | 222,281 | 4,334 | 46,222 | 454 | |

**Table S2**: Statistic summary of the overall fungal and bacterial sequences and operational taxonomic units (OTUs).

| Counts/sample summary | | | |
| --- | --- | --- | --- |
| Fungi | | OTU | |
| Total count | 1,960,578 | Total count | 1,825 |
| Min | 29,688 | Min | 272 |
| Max | 233,043 | Max | 733 |
| Median | 107,000 | Median | 499 |
| Mean | 109,000 | Mean | 488 |
| Std. dev. | 57,000 | Std. dev. | 128 |
|  |  |  |  |
| Counts/sample summary | | | |
| Bacteria | | OTU | |
| Total count | 3,045,338 | Total count | 11,560 |
| Min | 99,904 | Min | 2,186 |
| Max | 225,461 | Max | 4,484 |
| Median | 179,000 | Median | 3,360 |
| Mean | 169,000 | Mean | 3,290 |
| Std. dev. | 38,700 | Std. dev. | 706 |

**Tab. S3:** FDR-corrected *p*-values of the indicator bacterial genera and pairwise differences of log10 normalized abundances of genera in control and N-treated samples.

| Indicators bacterial genera | *p*-value | Pairwise differences |
| --- | --- | --- |
| *Bifidobacterium* | 0.007 | -1.109 |
| *Candidatus_Carsonella* | 0.02 | 1.063 |
| *Gilliamella* | 0.02 | -0.863 |
| *Lactobacillus* | 0.005 | -1.304 |
| *Leuconostoc* | 0.0002 | 1.937 |
| *Streptococcus* | 0.009 | -0.962 |
| *Zymobacter* | 0.01 | 1.014 |

**Tab. S4:** FDR-corrected *p*-values of the indicator fungal genera and pairwise differences of log10 normalized abundances of genera in control and N-treated samples.

| Indicators fungal genera | *p*-value | Pairwise differences |
| --- | --- | --- |
| *Curreya* | 0.01 | -0.827 |
| *Endomelanconiopsis* | 0.03 | -0.629 |
| *Hypotrachyna* | 0.02 | -1.036 |
| *Microcera* | 0.02 | 0.637 |
| *Naevala* | 0.01 | -0.677 |
| *Polyscytalum* | 0.006 | 1.801 |
| *Rhinocladiella* | 0.0005 | -1.296 |
| *Roussoella* | 0.006 | -0.887 |
| *Saccharomyces* | 0.03 | -0.817 |

**2. SUPPLEMENTARY FIGURES**

**
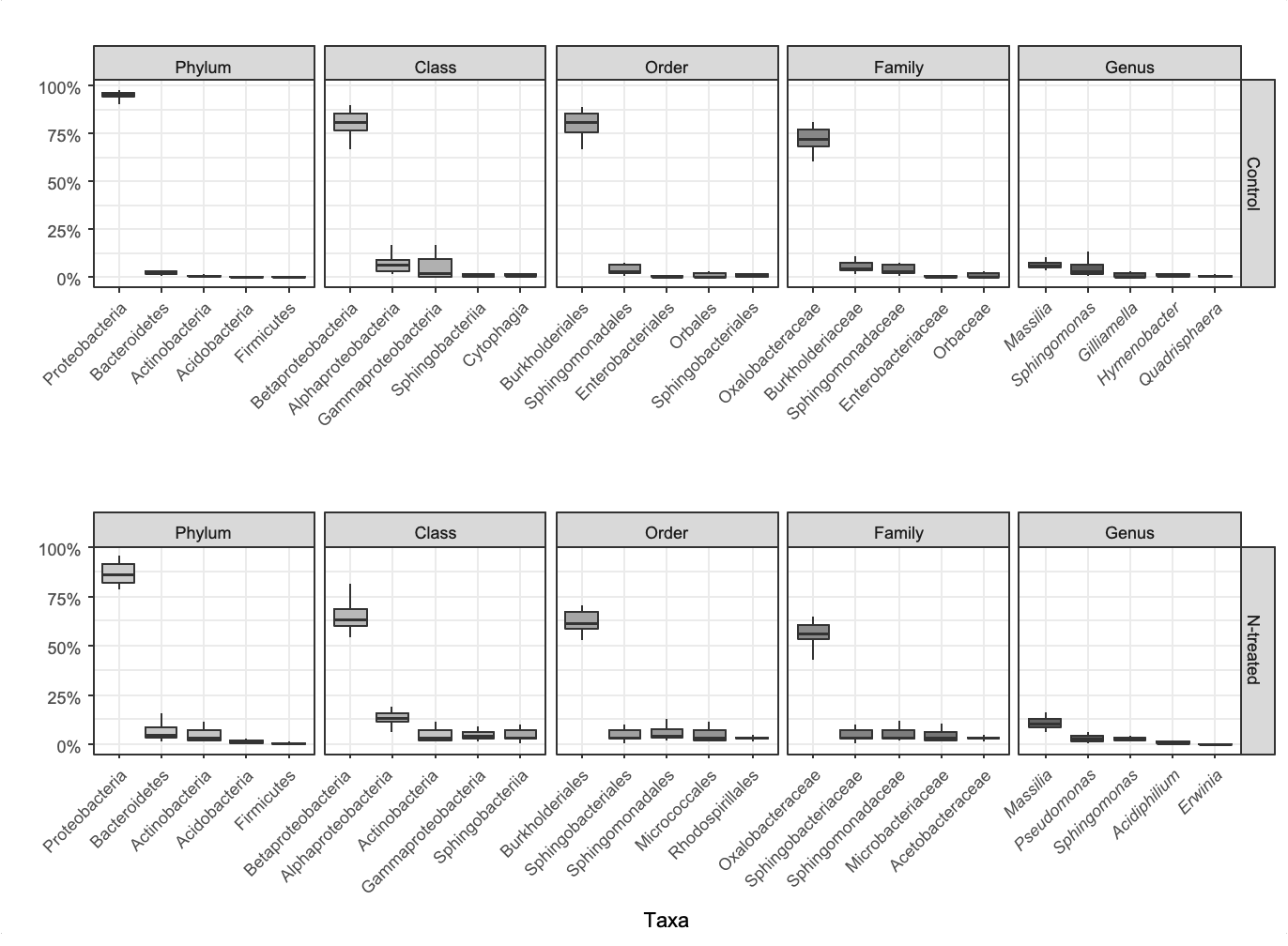
**

**Fig. S1A:** Relative abundance of top 5 bacterial taxa at each taxonomic rank in control and N-treated samples.

**
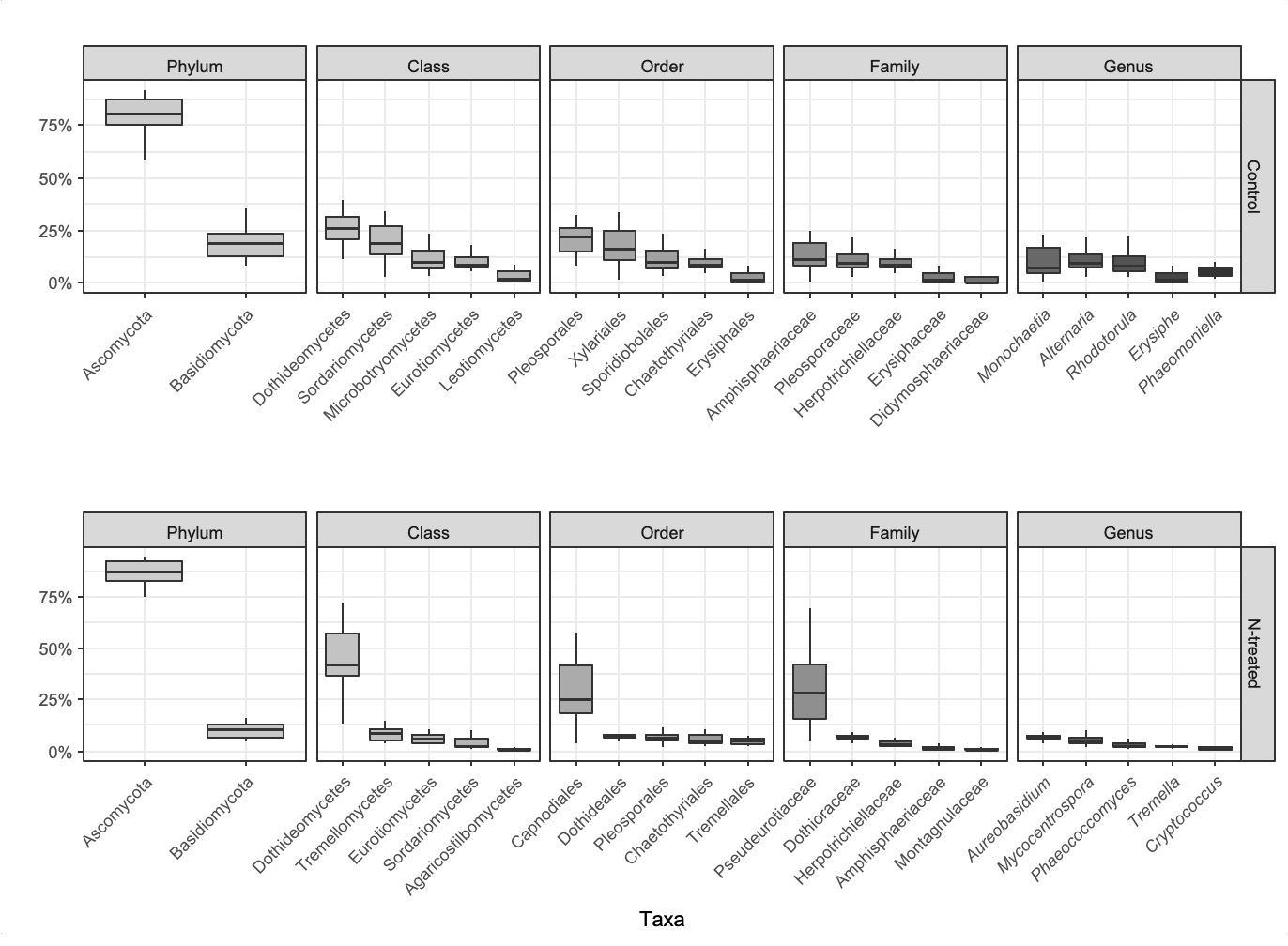
**

**Fig. S1B:** Relative abundance of top 5 fungal taxa at each taxonomic rank in control and N-treated samples. At the phylum level, only two taxa were found.

**
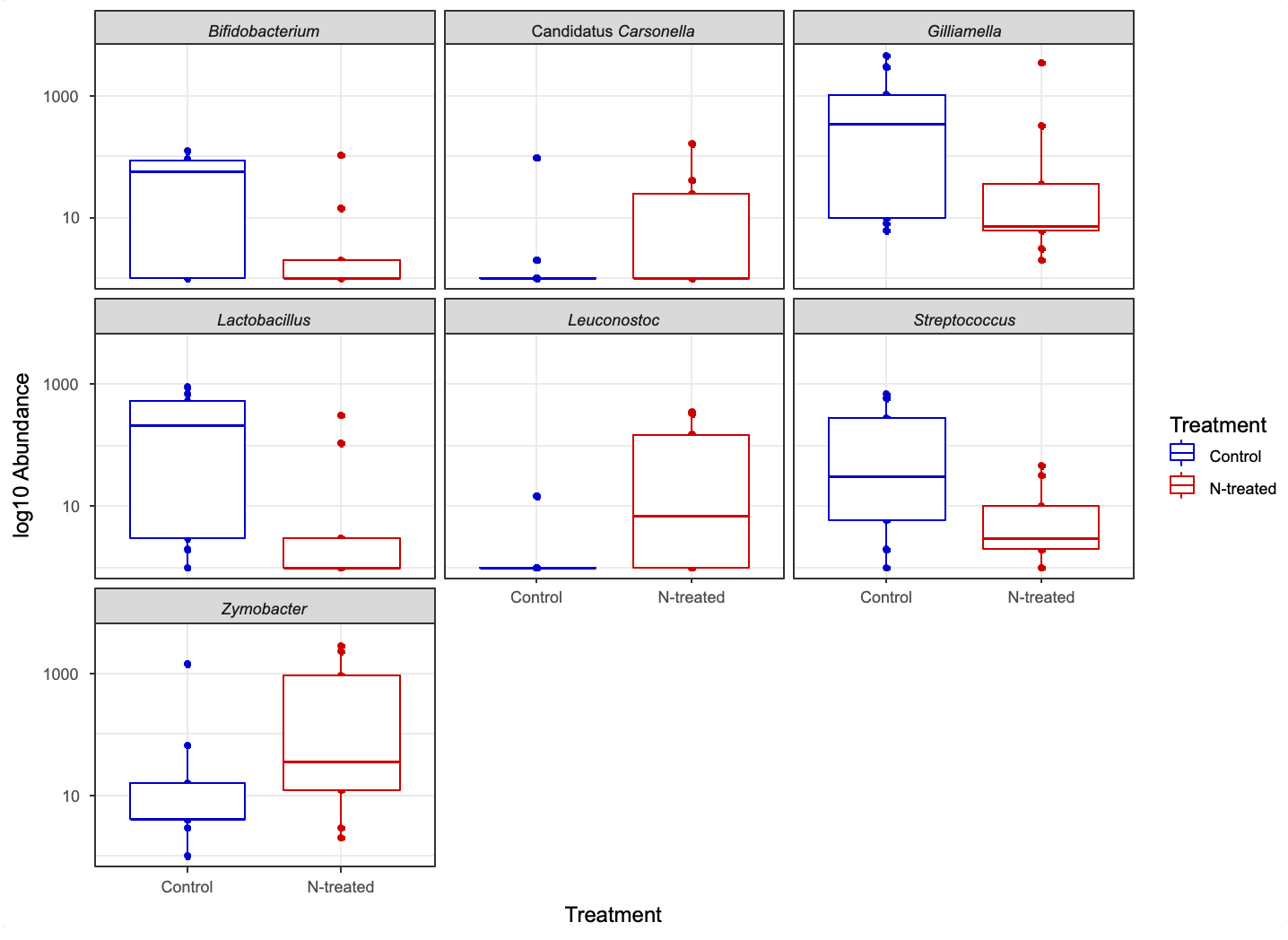
**

**Fig. S2 A:** Box plot representing the indicator bacterial genera for both control and N-treated samples. Only taxa statistically different between control and N-treated samples were plotted.

.


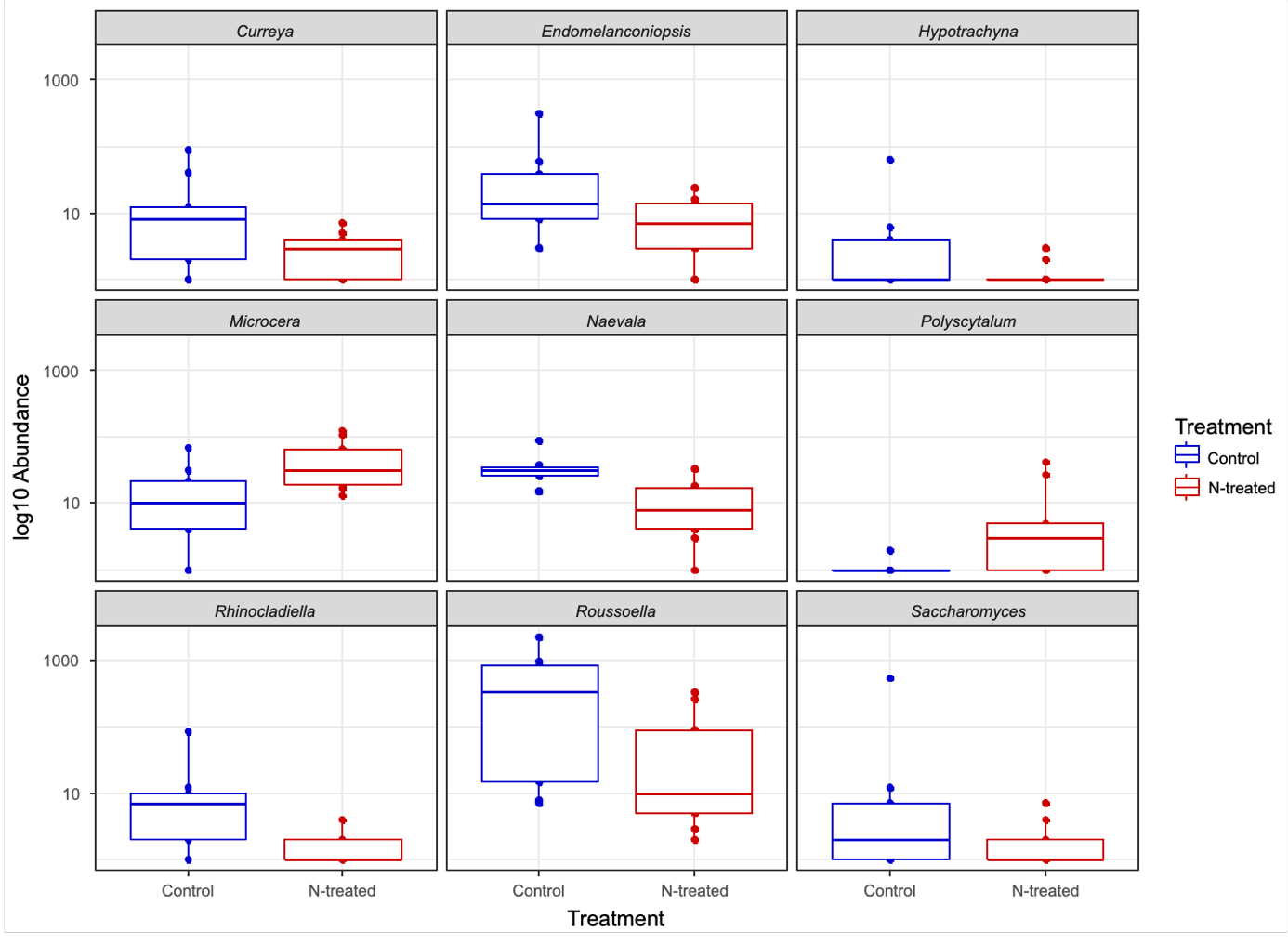


**Fig. S2 B:** Box plot representing the indicator fungal genera for both control and N-treated samples. Only taxa statistically different between control and N-treated samples were plotted.
